# Oral manifestations in a group of adults with autism spectrum disorder

**DOI:** 10.4317/medoral.17573

**Published:** 2011-12-06

**Authors:** Lorena M. Orellana, Francisco J. Silvestre, Sonia Martínez-Sanchis, Victoria Martínez-Mihi, Daniel Bautista

**Affiliations:** 1 DDS. Collaborator of the Red Cross Special Patients Clinic (Valencia); 2 Assistant Professor of Stomatology, University of Valencia. Head of the Stomatology Unit, Dr. Peset University Hospital (Valencia); 3 Assistant Professor of Psychobiology, University of Valencia; 4 Associate Professor of Stomatology, University of Valencia; 5 Staff physician, Department of Preventive Medicine, Dr. Peset University Hospital (Valencia), Spain

## Abstract

Objective: A number of studies have evaluated the oral health of patients with autism spectrum disorder (ASD), though most have involved children, and no specific oral manifestations have been described. The present study describes the buccodental disorders and hygiene habits in a group of adults with ASD. Study Design: A prospective case-control study was made of a group of patients with ASD (n=30), with a mean age of 27.7±5.69 years, and of a healthy age- and gender-matched control group (n=30). An evaluation was made of the medical history, medication, oral hygiene habits and oral diseases, with determination of the CAOD, CAOS and OHI-S oral hygiene scores. Results: Most of the patients in the ASD group used two or more drugs and were assisted in brushing 2-3 times a day. The most frequent manifestations were bruxism, self-inflicted oral lesions and certain malocclusions. The CAOD and CAOS scores were significantly lower than in the controls. Conclusions: Adults with ASD and assisted dental hygiene presented fewer caries than the non-disabled population. However, bruxism, ogival palate and anterior open bite were frequent in the patients with ASD.

** Key words:**Autism spectrum disorder, caries, dental hygiene, oral manifestations.

## Introduction

Patients with autism spectrum disorder (ASD) are the individuals with special needs that pose the greatest challenge for dentists, due to their complex and varied clinical manifestations.

Described by Leo Kanner in 1943, these manifestations first appear in individuals under three years of age, and are characterized by deficiencies in social interaction, communication, behavior, interests and activities (1), as well as complex sensory alterations (2).

Management is of a multidisciplinary nature, the most effective strategies being based on educational programs (3). In order to educate children with ASD, it is necessary to facilitate structured situations helping the patients to anticipate what is going to happen (4), since they show great fear and anxiety in the face of unknown situations (5), such as visiting the dental clinic. The dental care of these patients poses great difficulties, and in most cases treatment is provided under general anesthesia (4,6-8).

No specific oral manifestations of ASD have been described (1,7,9), though the oral hygiene of these subjects is known to be deficient (1,5,10,11). Nevertheless, many authors have found the prevalence of caries and of periodontal disease to be no different compared with non-autistic individuals (4,5,9,10), and in some cases the prevalence of caries in children with ASD may even be comparatively lower (6,8,12). Between 60-95% of all patients with ASD have an unusual sensory profile, including dysfunction in registering oral sensitivity.

Most studies of patients with ASD report oral pathology in children, and in some isolated instances both children and adults have been evaluated. Very few studies have examined only adults with ASD, and no case-control series have been published. The present study was therefore designed to describe the habits of oral hygiene and buccodental disease in a group of adults with ASD in our community.

## Material and Methods

Study design

A prospective case-control study was carried out, describing the habits of oral hygiene and oral pathology in a group of adults with ASD, compared with a group of healthy age- and gender-matched controls.

Sample screening

The study population consisted of individuals with ASD pertaining to two day centers for people with autism in the Valencian Community (Spain) (one in the province of Castellón and the other in the province of Valencia).

Of the 40 individuals in these centers, 30 met the study inclusion criteria. The latter were defined before sample screening and consisted of the following: a diagnosis of ASD; the understanding at least of very simple instructions; and the obtainment of written informed consent from the caregivers for participation in the study.

The study sample consisted of 27 males and 3 females (n=30), aged between 20-41 years (mean 27.7±5.69). All patients presented some degree of mental impairment: mild in 8 cases (26%), moderate in 11 (37%) and severe in 11 (37%). A total of 63.33% of the patients were institutionalized.

The control group in turn consisted of 30 non-disabled individuals randomly selected from among the accompanying persons in the Red Cross Special Patients Clinic of Valencia (Spain): 23 males (76.67%) and 7 females (23.33%), with a mean age of 27.83±5.84 years.

Methodology

The parents / caregivers of the participants received an explanation of the study, together with an information sheet and informed consent form. Those who agreed to participate completed a questionnaire evaluating the medical history and habits of oral hygiene of the patients. The data not adequately recorded in this way were obtained by interviewing the parents / caregivers. The study received the support and collaboration of the technical personnel of the different participating centers. Two dentists specialized in the oral care of disabled patients participated, together with a stomatologist and a psychologist specialized in ASD.

One to 5 anticipatory workshops or sessions were held per subject before the dental examination. To this effect we used photographs, pictograms, macro models, tooth brushes and real objects such as caries probes, dental mirrors, masks and gloves. Each workshop lasted 20 minutes, with the participation of 1-2 people. Desensitization was achieved through successive approaches, and use was made of the Tell-Show-Do (TSD) technique, visual pedagogy, in vivo modeling, audiovisual modeling, behavioral testing and self-modeling from photographs. Sensory processing was assessed with the widely validated Sensory Profile Questionnaire. The sensory profile reflects the response to sensory stimuli. In this way we can identify the search for sensations, emotional reactivity, tone / resistance, oral sensory sensitivity, inattention / distractibility, poor registry, sensory sensitivity, sedentarism and fine motor perception.

All clinical dental examinations were made by the same dentist. A number 5 dental mirror was used, together with a caries exploratory probe. The exploration was carried out in an adequately conditioned room with limited decoration, silence and no distractions. A stretcher-type chair and guidable light source of sufficient power for adequate intraoral exploration were used, and all the data were recorded in the patient clinical history.

At the end of the examination, the parents / caregivers received a report on the oral disorders found, with suggestions regarding the necessary treatments and dental care.

The CAOD index was determined in both groups, assessing the carried, absent and obturated teeth corresponding to all the individuals examined per group (considering only 28 permanent teeth). The CAOS index was also determined under the same conditions as before, though in this case the basic unit was the dental surface. Specifically, 5 surfaces were considered in posterior teeth, and four surfaces in anterior teeth. Oral hygiene was rated using the Simplified Oral Hygiene Index (OHI-S). The criteria of this index are related to the sum of the Plaque Index Score (PI-S) and the Calculus Index Score (CI-S). In both cases the surfaces of index teeth were examined: vestibular surfaces of 11, 16, 26 and 31; lingual surfaces of 36 and 46. In the absence of index teeth, we explored the entire sextant and recorded the maximum degree (except third molars).

The PI-S was scored as 0: no plaque; 1: plaque covering no more than 1/3 of the examined surface; 2: > 1/3 but < 2/3; 3: > 2/3 of the surface. The CI-S was scored as 0: no calculus; 1: supra gingival calculus < 1/3; 2: >1/3 but <2/3 of the surface or isolated sub gingival points; 3: > 2/3 of the surface or sub gingival in the form of a continuous band.

Data analysis

The SPSS version 18 statistical package for Microsoft Windows® was used to analyze the data. A descriptive study was made involving the frequencies of the different variables, together with a comparative analysis of the two groups.

The Student t-test was used to compare means of quantitative variables between the two groups, while the Mann-Whitney U-test was used for continuous / discontinuous variables showing a non-normal (skewed) distribution. The Fisher test was used for comparing qualitative variables. Statistical significance was accepted for p<0.05 and p<0.01.

Clinical research ethics committee

The study was evaluated and approved by the Clinical Research Ethics Committee of the University of Valencia.

## Results

The medical history of the patients with ASD revealed only two cases (6.67%) of gastroesophageal reflux, and 5 cases (16.67%) of epilepsy. Fifty percent suffered self-inflicted lesions (80% affecting the hands and arms, 47% the head and neck, and only 13% the rest of the body). A full 77% were receiving some kind of medication. The drug distribution was as follows: anxiolytics 57%, anti psychotics 48%, anticonvulsivants 39%, neuroleptics 22%, antidepressants 17%, and other medications 22%. Of the 23 patients under medication, 78% received two or more drugs.

As regards oral hygiene, almost one-half of the patients with ASD (46.67%) underwent fully assisted tooth brushing. Thirty percent were initially autonomous, but finally required the help of the parents / caregivers. Only 23.33% were fully autonomous for tooth brushing. In turn, 46.67% brushed at least three times a day, 40% twice a day, and 13.33% once a day. Only a manual toothbrush was used in 25 cases (83.33%), and an electric toothbrush in 5 cases (16.67%).

Extra oral examination of the patients with ASD revealed cheilitis in 13.33% of the cases and drooling in 7%. Intraoral examination in turn revealed self-inflicted lesions in 13% of the cases, corresponding to biting of the lips and inside of the cheek, and intraoral ulcers. Over one-half of the patients (60%) showed wear facets, affecting the enamel and dentin in 61% of the cases. Twenty percent suffered dental traumatisms – all in the upper anterior sector. Regarding the presence of malocclusions, 36.67% presented ogival palate, 46.67% dental crowding, and 30% anterior open bite.

Caries were present in 60% of the subjects. The CAOD score for the 30 patients was 3.7, with values of 1.33, 1 and 1.37 for caried, absent and obturated teeth, respectively. The OHI-S score was 1.92, and the PI-S score (1.57) was greater than the CI-S score (0.41).

On comparing oral hygiene among the patients with ASD and the controls, only 23.33% of the former were seen to brush their teeth autonomously, versus 100% of the subjects without ASD. In most of the patients with ASD the brushing frequency was at least three times a day, versus twice a day in the controls ( Table 1).

**Table 1:**
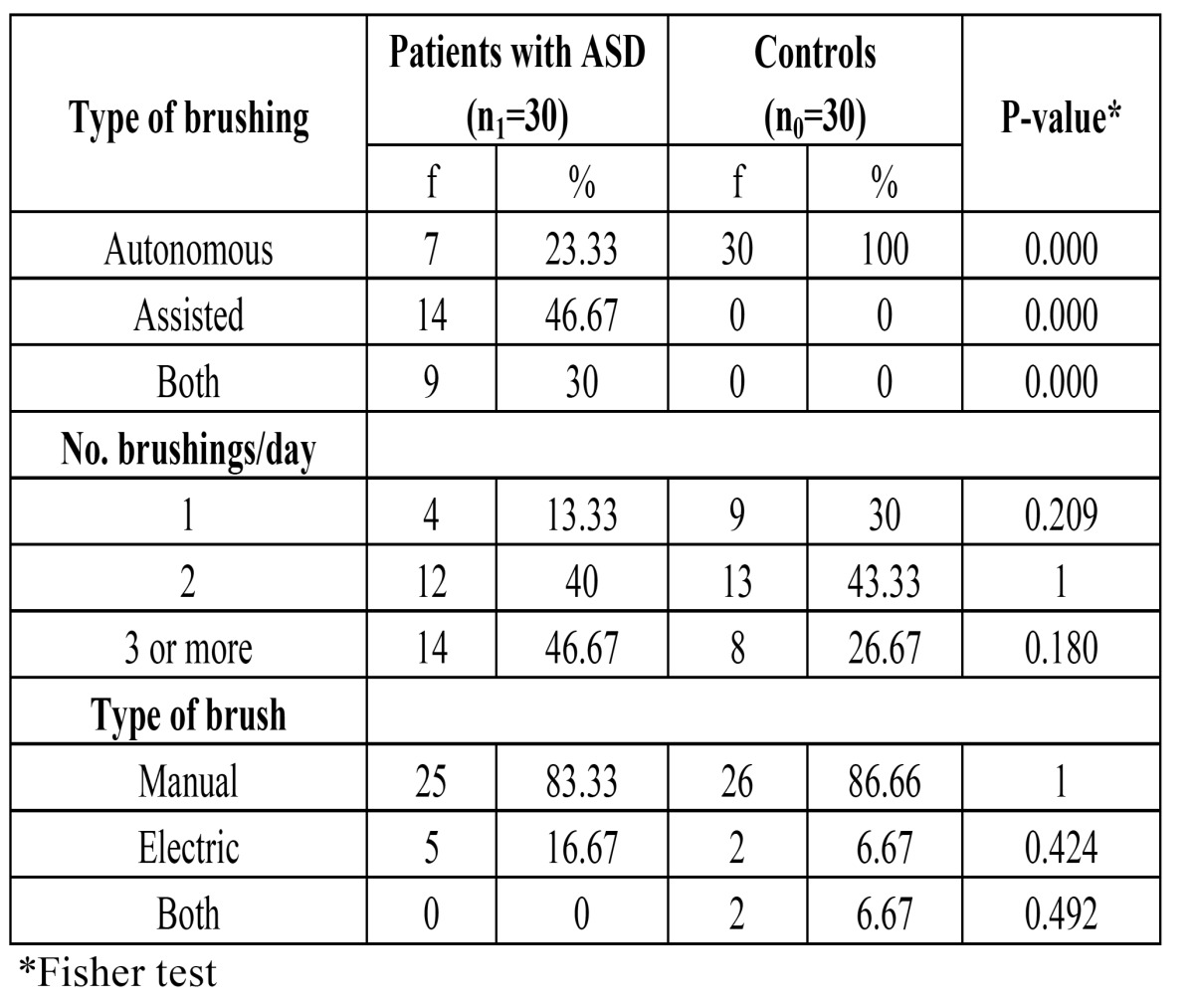
Comparison of oral hygiene between patients with ASD and the controls.

On comparing the oral pathology in the two groups, a significantly greater presence of ogival palate and anterior open bite was recorded in the ASD group (p<0.05 and p<0.01, respectively) ( Table 2).

**Table 2:**
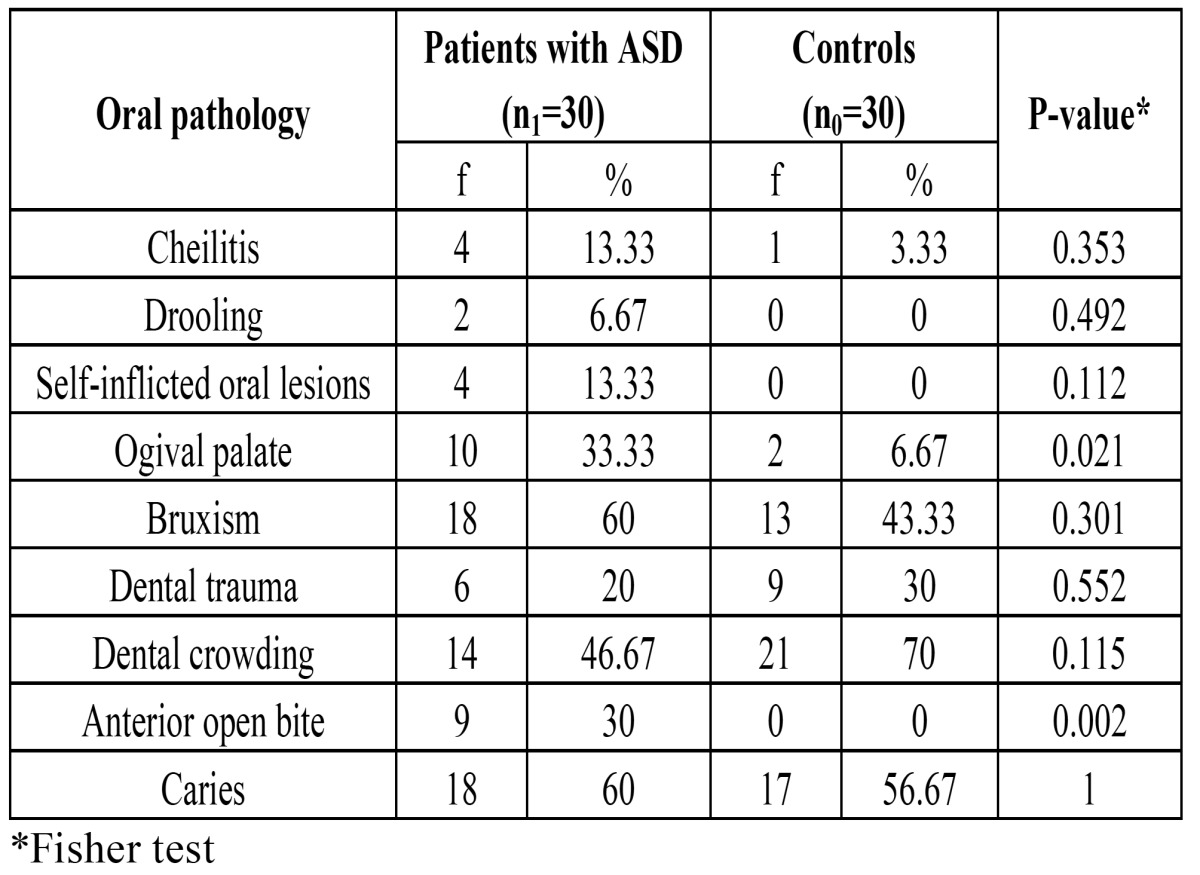
Comparison of oral pathology between patients with ASD and the controls.

( Table 3) shows the caries and oral hygiene index scores for both groups. Statistically significant differences were observed in relation to the CAOD index (p<0.05) and the number of obturated teeth (p<0.01) and surfaces (p<0.05). Regarding the obturated teeth, the ASD group presented an average of 0.97 teeth filled with amalgam and 0.40 with resin composite, versus 0.73 and 2.47 in the control group, respectively (p<0.01). In the ASD group 2.44% of the patients had anterior teeth filled with resin composite, versus 3.8% in the control group.

**Table 3:**
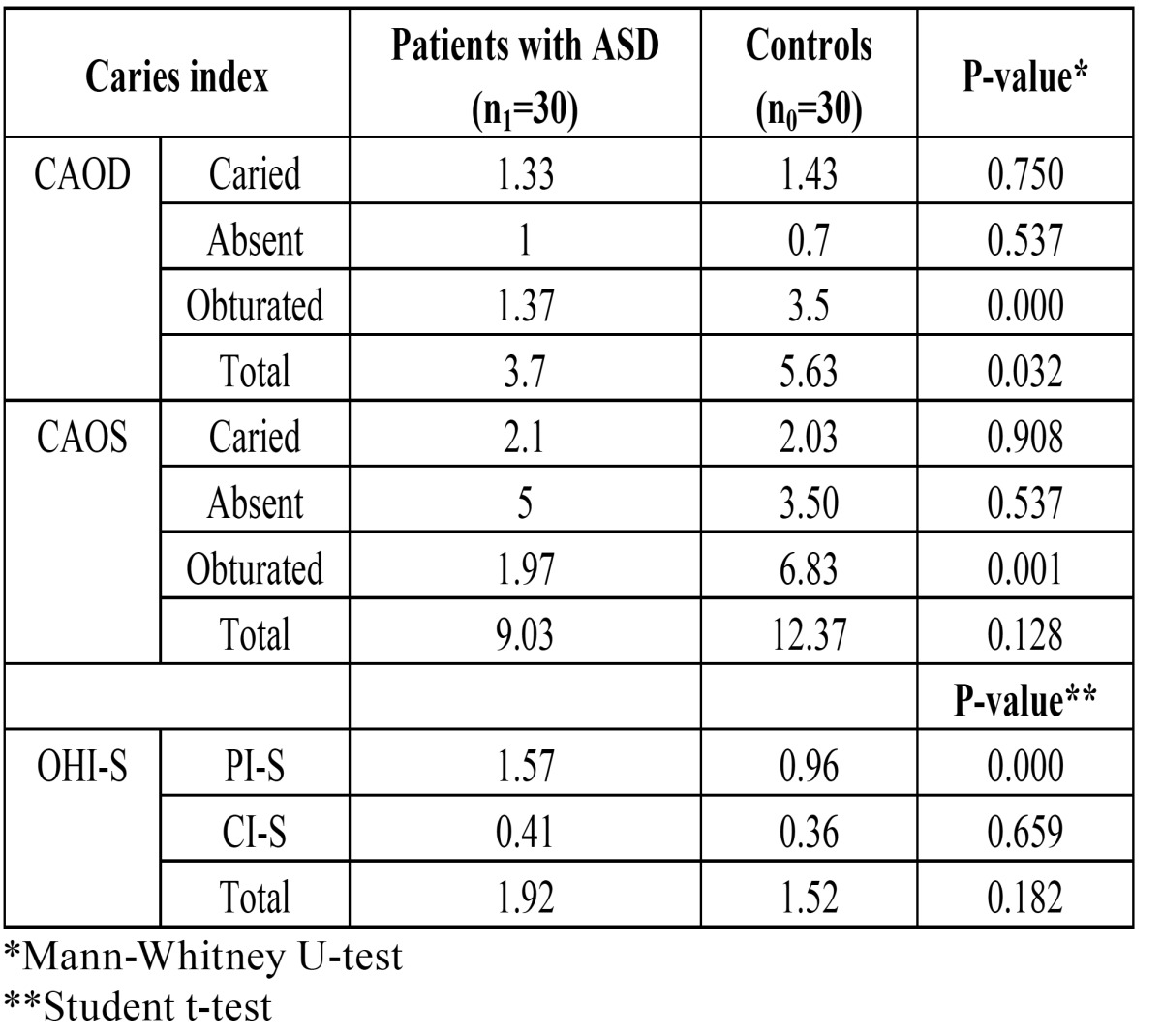
Comparison of caries index and oral hygiene between patients with ASD and the controls.

The amount of plaque was significantly greater in the ASD group than in the controls without ASD (p<0.01).

The time taken to complete the dental examination in the ASD group was 8.13 minutes (range 5.33-15.5). In comparison, the time taken to complete the dental examination in the control group was 5.52 minutes (range 4.4-6.2) – the inter group difference being statistically significant (p<0.01). One half of the patients with ASD showed altered processing in various sensory modalities – this evidencing the presence of a global sensorial disorder. Inadequate oral sensitivity registry was documented in 25% of the cases – a situation that could have influence the habits of oral hygiene and oral disease in the study sample.

## Discussion

The medical history of patients with autism spectrum disorder (ASD) could condition the appearance of certain oral lesions or may influence the definition of a dental treatment plan. In our study there were fewer patients with gastroesophageal reflux than in other studies such as that published by Ibrahim et al. (13). Likewise, we observed a lesser prevalence of epilepsy (9,14). However, self-inflicted injuries are one of the manifestations that may pose a problem for the dental management of these patients. The prevalence of these lesions was 13% in our series – a proportion similar to that reported by Baghdadli et al. (15), but far lower than in other studies (in the order of 68%) (6,16).

Most of the patients with ASD were receiving drug treatment, and a large percentage received 2-3 drugs a day. These values are far higher than those reported by Loo et al. (12), and may be due to the fact that our series involved only adult patients, most of which were moreover institutionalized, while most other studies found in the literature are about pediatric patients. The most frequently prescribed drugs were anti psychotics and anxiolytics that can affect salivation and favor bacterial plaque formation and caries. Nevertheless, we found no differences between the two groups in terms of the presence of caries.

Most of the patients with ASD required assistance in tooth brushing, in coincidence with the observations of other authors (9,17,18). Indeed, only one-quarter of the patients were able to complete brushing without help. However, the frequency of assisted brushing was high (2-3 times a day), and mostly involved manual brushing rather than the use of an electric toothbrush (6,17). This may be explained by the fact that most of the patients were institutionalized adults following a habit acquired with their caregivers.

In any case, dental hygiene tends to be insufficient due to dietary problems, since these patients show a preference for certain flavors and textures (e.g., sweet, soft and sticky foods). Another contributing factor may be food packing in the mouth for a period of time, together with dependency on other people for keeping adequate oral hygiene.

The studies carried out to date on the impact of caries in patients with ASD have involved children or very heterogeneous groups of individuals with a broad age range. The only study on the prevalence of caries in adults with ASD has been published by Shapira et al. (5), involving a group of 17 institutionalized individuals with a mean age of 22 years – the recorded CAOD and CAOS scores being 7.11 and 16.12, respectively, i.e., almost double the scores obtained in our series. Furthermore, we observed no differences versus the control group in terms of the caries component of the CAOD index. This would reflect the efficacy of increasing the frequency and efficiency of assisted brushing in this group of disabled patients. In contrast, significant differences were observed in the case of the complete CAOS index and the scores by surfaces, which were both lower in the ASD group.

On examining the number of dental traumatisms, our data coincide with those found in the literature (20% and 21%) (9,19). All of these injuries affected teeth in the anterior sector, and were due to falls secondary to walking problems and the lack of patient caution in situations of risk – which increases vulnerability to accidents.

Patients with ASD do not present very specific oral disorders (1,7,9), though they do show a greater tendency towards certain malocclusions (e.g., ogival palate and anterior open bite). Ozgen et al. (20) also observed these differences regarding malocclusion. Likewise, our patients with ASD showed dental crowding in a larger proportion of cases than in the study of De Mattei et al. (21), though the prevalence was comparatively higher in the control group.

Bruxism was more frequent than in other studies (21% to 44%) (6,19,21). This may be because the mentioned studies evaluated mainly children, and the patients with bruxism in our study were institutionalized and presented important mental retardation. Lastly, while we also recorded self-inflicted oral lesions and drooling, the proportions were lower than in other studies (21).

In conclusion, the present study shows that institutionalized adults with ASD and assisted dental hygiene present fewer caries than the non-disabled population. The patients do show some characteristic oral manifestations in the form of self-inflicted lesions, bruxism and malocclusions such as ogival palate and anterior open bite.

## References

[1] Klein U, Nowak AJ (1998). Autistic disorder: a review for the pediatric dentist. Pediatr Dent.

[2] Corbett BA, Schupp CW, Levine S, Mendoza S (2009). Comparing cortisol, stress, and sensory sensitivity in children with autism. Autism Res.

[3] Ferrando-Lucas MT, Martos-Pérez J, Llorente-Comí M, Freire-Prudencio S, Ayuda-Pascual R, Martínez Díez-Jorge C (2002). The autistic spectrum. An epidemiological study and analysis of possible subgroups. Rev Neurol.

[4] Swallow JN (1969). The dental management of autistic children. Br Dent J.

[5] Shapira J, Mann J, Tamari I, Mester R, Knobler H, Yoeli Y (1989). Oral health status and dental needs of an autistic population of children and young adults. Spec Care Dentist.

[6] Kamen S, Skier J (1985). Dental management of the autistic child. Spec Care Dentist.

[7] Dávila JM, Jensen OE (1988). Behavioral and pharmacological dental management of a patient with autism. Spec Care Dentist.

[8] Namal N, Vehit HE, Koksal S (2007). Do autistic children have higher levels of caries? A cross-sectional study in Turkish children. J Indian Soc Pedod Prev Dent.

[9] Klein U, Nowak AJ (1999). Characteristics of patients with autistic disorder (AD) presenting for dental treatment: a survey and chart review. Spec Care Dentist.

[10] Lowe O, Lindemann R (1985). Assessment of the autistic patient’s dental needs and ability to undergo dental examination. ASDC J Dent Child.

[11] Bäckman B, Pilebro C (1999). Visual pedagogy in dentistry for children with autism. ASDC J Dent Child.

[12] Loo CY, Graham RM, Hughes CV (2008). The caries experience and behavior of dental patients with autism spectrum disorder. J Am Dent Assoc.

[13] Ibrahim SH, Voigt RG, Katusik SK, Weaver AL, Barbaresi WJ (2009). Incidence of gastrointestinal symptoms in children with autism: a population-based study. Pediatrics.

[14] Tuchman R, Cuccaro M, Alessandri M (2010). Autism and epilepsy: historical perspective. Brain Dev.

[15] Baghdadli A, Pascal C, Grisi S, Aussilloux C (2003). Risk factors for self-injurious behaviours among 222 young children with autistic disorders. J Intellect Disabil Res.

[16] Rodríguez-Abellán J (1999). Therapeutic intervention in infantile autism and generalized developmental disorders: self-injury and selfstimulation. Rev Neurol.

[17] Marshall J, Sheller B, Mancl L (2010). Caries-risk assessment and caries status of children with autism. Pediatr Dent.

[18] Marshall J, Sheller B, Williams BJ, Mancl L, Cowan C (2007). Cooperation predictors for dental patients with autism. Pediatr Dent.

[19] Kopycka-Kedzierawski DT, Auinger P (2008). Dental needs and status of autistic children: results from the National Survey of Children’s Health. Pediatr Dent.

[20] Ozgen H, Hellemann GS, Stellato RK, Lahuis B, van Daalen E, Staal WG (2011). Morphological features in children with autism spectrum disorders: a matched case-control study. J Autism Dev Disord.

[21] DeMattei R, Cuvo A, Maurizio S (2007). Oral assessment of children with an autism spectrum disorder. J Dent Hyg.

